# Replacing serum with dried blood microsampling for pharmacokinetics, viral neutralisation and immunogenicity bioanalysis supporting future paediatric development of RSM01, a candidate respiratory syncytial virus neutralising monoclonal antibody

**DOI:** 10.1186/s12879-024-10196-4

**Published:** 2024-12-18

**Authors:** Joleen T. White, Jonne Terstappen, Micha Levi, Andrijana Radivojevic, Robert Noble, Aparna B. Anderson, Gwendolyn Wise-Blackman, Michael W. Dunne

**Affiliations:** 1 Bill & Melinda Gates Medical Research Institute, Cambridge, MA USA; 2 Center for Translational Immunology, University Medical Centre Utrecht, Utrecht, The Netherlands; 3 intiGROWTH LLC, Miami, FL USA; 4 BioData Solutions, Lawrence, KS USA

**Keywords:** Patient-centered, Dried blood, Monoclonal antibody, Neutralising antibodies, Viral neutralising activity, Pharmacokinetics, Respiratory syncytial virus, Volumetric absorptive microsampling, Antidrug antibodies, Prophylaxis

## Abstract

**Background:**

Virus neutralising antibodies in serum are considered key correlates of protection for vaccines and monoclonal antibodies against respiratory syncytial virus (RSV). RSM01 is a novel, highly-potent, half-life-extended and fully-human monoclonal antibody candidate targeting the RSV prefusion F protein. Currently in Phase 1 development, RSM01 is primarily being developed to potentially provide an effective and affordable RSV prevention strategy in low- and middle-income countries. To evaluate the ability of dried blood collection to generate data sets and conclusions comparable to serum collection, we compared pharmacokinetics (PK) of RSM01, immunogenicity, and virus neutralisation for dried capillary blood samples with serum samples.

**Methods:**

RSM01 PK, anti-drug antibodies (ADA), and RSV-neutralising antibodies from the Phase 1 trial were analyzed and compared between matched serum and dried blood samples. Deming regression analysis was performed using baseline-corrected values to evaluate correlation between measurements in liquid serum versus dried blood.

**Results:**

The analysis showed good correlation (R^2^ > 0.95) between individual RSM01 concentrations measured in both serum and capillary blood. Analysis of RSM01 PK parameters in capillary blood yielded equivalent conclusions as from serum. A strong correlation (R^2^ > 0.95) was observed between RSV neutralising activity measured in both serum and capillary blood. In addition, RSV neutralising activity was correlated with RSM01 concentrations in both serum and capillary blood data sets. For ADA, individual sample results had 96% agreement (290/302) and overall participant ADA status had 93% agreement (52/56).

**Conclusions:**

Both RSM01 concentrations and RSV neutralising activity showed a strong correlation between the serum and blood measurements. ADA measurements also had an agreement of > 90% for individual samples and overall participant status. Our results demonstrate that dried blood is a suitable specimen type for collection and evaluation in the RSM01 clinical development program and shows promise as a useful approach to reduce patient burden in clinical trials, particularly for infants in low- and middle-income countries.

**Trial Registration:**

Clinicaltrials.gov NCT05118386 November 12, 2021.

**Supplementary Information:**

The online version contains supplementary material available at 10.1186/s12879-024-10196-4.

## Background

Respiratory syncytial virus (RSV) is the leading cause of acute lower respiratory tract infection (LRTI) in infants and young children worldwide, particularly in low- and middle-income countries (LMICs) [1]. Current approaches to prevent RSV disease include vaccination of pregnant individuals and passive immunization with monoclonal antibodies (mAbs) [2]. RSM01 is a novel, highly potent, fully human, RSV-neutralising mAb candidate with an extended half-life and is being developed primarily for LMICs [3].

Given that the clinical development of RSM01 focuses on the infant population experiencing their first RSV season [4], we considered aspects of patient-centricity within the RSM01 clinical development program. Two key aspects to patient-centricity within the bioanalytical sciences include (1) optimal sample collection to make sure that every sample being collected has a clear use and (2) minimizing sampling requirements (both number of samples and volume of samples) for trial participants [5]. Reducing sample volumes is particularly important for infant populations due to lower weight and blood volume, as oversampling can lead to iatrogenic anemia [6].

While one approach is to reduce sample collection volume while maintaining traditional venipuncture techniques, this still requires personnel trained in venipuncture, on-site or nearby equipment to process the venous blood to serum, and cold chain storage and shipment of the serum samples. The venipuncture procedure itself is particularly challenging in patients with difficult venous access, for which risk factors in children include younger age, female sex, darker skin color, prematurity, and sickle cell disease [7, 8]. As these factors align with our target population of infants in LMIC, capillary collection was evaluated as a preferred approach to both reduce sample volume and the impact of the venipuncture procedure in vulnerable populations.

Dried blood spot (DBS) sample collection is the most well-known and researched form of microsampling, with multiple applications across different analytes including nucleic acids, peptides and proteins [5, 9, 10]. DBS is one of the least invasive blood sample collection methods, with samples easily transported and stored [11, 12]. One of the most common and widely accepted clinical uses of DBS sampling is in neonatal screening programs. DBS has also been used to assess infectious disease serology from both natural immunity and vaccines [10, 13, 14]. While these serology assays have been conducted as binding assays rather than functional assays, other functional enzyme assays have previously been assessed successfully by DBS [15].

Development of volumetric based dried blood collection has expanded the use of dried blood to now include quantitative drug analysis applications, including therapeutic antibodies [16–19]. A key advantage of volumetric sampling versus DBS is precise collection of a fixed volume of blood rather than a fixed diameter of dried blood, which reduces variability due to haematocrit viscosity effect impacting spread of DBS [20, 21]. Several of the volumetric sampling devices also employ a polymeric substrate, which has been helpful to facilitate complete recovery of hydrophilic antibodies without requiring the use of additional steps to disrupt the paper substrate of DBS [17, 18, 22]. The specific technology evaluated in this work was volumetric absorptive microsampling (VAMS), which also has the advantage of using capillary action to wick up the blood from the finger stick or heel stick directly to minimize collection volume [17, 18, 23].

In conjunction with the advances in microsampling has come increased familiarity and acceptance of these approaches by health authorities. Validation of methods on dried matrices is included in ICH M10 [24, 25]. If data from both serum and blood specimens are used within a program, a bridging experiment is required, however it does not require nearly as many samples as the fully matched trial design conducted herein.

Since the RSM01 development program targets an infant population with the eventual goal of global enrollment at a variety of clinical sites with different resource constraints, we decided to use small volumes of capillary blood as the primary specimen type for the entire RSM01 clinical development program. As such, we took the opportunity in the first in human (FIH) trial Gates MRI-RSM01-101 to collect both capillary blood and venous serum for the bioanalytical program. The analytes tested included RSM01, anti-drug antibodies (ADA) against RSM01, and RSV-neutralising activity whether facilitated by RSM01 or the result of natural immunity. We selected the specific cell-based RSV neutralisation assay for the low sample volume required, the short incubation time, the relatively high throughput, and use of common instrumentation [26].

Within the Gates MRI-RSM01-101 trial, we did fully matched sample collection in this population of healthy adults to expand analyses beyond correlation of individual sample results to also enable comparison between conclusions derived from the pharmacokinetics (PK) analysis. While this duplicated sample collection design far exceeds the requirements of a bridging trial and is not patient-centric for the participants in Gates MRI-RSM01-101, the patient-centric focus was targeted towards the infant populations planned for enrollment in the subsequent development program of RSM01.

This manuscript describes the results of the exploratory objective in Gates MRI-RSM01-101 to evaluate the feasibility of using capillary blood in future RSM01 clinical development that will be conducted entirely in infants.

## Methods

### First-in-human trial

The first-in-human, double-blind, phase 1 trial of RSM01 enrolled healthy adults of 18–49 years of age [4]. In brief, participants were randomised 6:1 to receive a single dose of RSM01 or placebo in dose ascending cohorts of 300 mg intravenously (IV), 300 mg intramuscularly (IM), 1000 mg IV, 3000 mg IV, or in a dose expansion cohort of 600 mg IM. The primary objective was to evaluate the safety of a single dose of RSM01. Secondary objectives included characterization of the PK and anti-drug antibodies. Exploratory objectives included characterization of RSV-neutralising antibodies as well as comparison of PK and pharmacodynamics between serum and capillary blood samples.

Serum and blood samples were collected at fully matched time points through 151 days (Supplement Table 1). Serum samples were collected by venipuncture in serum separator tubes. Dried blood samples from finger sticks were collected by touching the Mitra VAMS (Trajan Scientific, Australia) from above the blood spot to wick up the fixed volume. Both serum and VAMS samples were stored at -70 °C and shipped on dry ice.

### RSM01 concentration

Separate validated immunoassays were used for the quantification of RSM01 in serum and in dried blood extract. In summary from full method (Supplemental Methods), dried blood was extracted from the 20 µL VAMS device into 200 µL buffer. Calibrators, quality control (QC) samples, and clinical trial samples (serum or extracted blood in separate assays) were diluted to the minimum required dilution (MRD) and captured on coated MSD plates. After incubation and washing, detection antibody conjugated to Ruthenium (MSD Sulfo Tag Gold, Mesoscale Discovery, Gaithersburg, MD, USA) was added to the plate and incubated. Plates were then washed followed by the addition of MSD Read Buffer. Relative light units (RLU) were captured on an MSD Sector 600 Imager. The concentration of RSM01 was calculated from a 5-parameter nonlinear regression curve with 1/Y^2^ weighting of calibrators.

### PK non-compartmental analyses

The PK analysis included all participants who received RSM01 and had baseline and at least one post-baseline serum or blood PK result. PK parameters were determined using noncompartmental analysis (NCA) of concentration-time data performed with Phoenix^®^ WinNonlin^®^ v.8.4 PK parameters analysed included maximum drug concentration (C_max_), time to C_max_ (T_max_), and area under the curve up to the last measurable concentration (AUC_last_) in serum and blood. Some PK parameters (AUCinf, CL, CL/F, Vz, Vz/F and t1/2) were not calculated due to unreliable terminal phase (AUC extrapolation > 20%).

### RSV-neutralising antibodies

Separate qualified viral neutralising assays were used to determine neutralising capacity as described previously [27]. In brief, 20µL of dried blood was eluted overnight at room temperature into 200 µL PBS. Both serum and dried blood eluate were heat-inactivated at 56 °C for 30 min before serial dilution in medium. Recombinant mKate-RSV-A2 [28] was added 1:1 to the sample dilution series and incubated for 1 h at 37 °C before addition of 50 µL of sample-virus mixture to a monolayer of Hep-2 cells. After 26 h incubation, fluorescence was recorded and 50% inhibitory dilution (ID50) was calculated from a 4-parameter nonlinear regression curve.

### Anti-drug antibodies

Separate validated bridging immunoassays were used for the detection of anti-drug antibodies (ADA) against RSM01 in serum and in dried blood extract. In summary from full method (Supplemental Methods), dried blood was extracted from the 20 µL VAMS device into 200 µL buffer. Samples were diluted to the MRD and incubated with biotin-labelled RSM01 and ruthenium-conjugated RSM01. Complexes of labeled RSM01 and ADA were captured on streptavidin MSD plates. Samples above the threshold in the screening assay were subsequently analysed in the confirmatory assay to evaluate a drop of signal in the presence of excess unlabeled RSM01. Samples that were positive in the confirmatory assay were titrated by serial dilution to determine the reciprocal value of the highest dilution factor considered positive.

Overall ADA status was derived across the individual results for each participant. For the agreement analysis, participants were considered positive if they had any ADA positive samples, whether pre- or post-baseline.

### Correlation analyses

Correlation analysis was performed using Deming regression [29], which minimizes the perpendicular distances between the data points and the regression line, thus incorporating known variability in measuring both variables. The variance ratio was set to one since both variables had similar value ranges, making the Deming regression equivalent to an orthogonal regression. Additional discussion on selection of Deming regression is in the Supplemental Methods.

Regressions were performed primarily on the PK population, except for comparing the RSV neutralising activity between serum and blood, which was performed on the per protocol population. The per protocol population included all participants who did not have a significant protocol deviation. The PK population included all participants who received RSM01 and had a baseline and at least one post-baseline PK assessment.

Due to the wide range of values within the regression and estimated errors being proportional to values rather than fixed across the range, the Deming regressions were performed on natural log transformed data. For RSM01 concentrations, the natural log was performed on ng/mL units. The regression lines were then converted to exponential lines for plotting with raw data.

## Results

We measured three types of antibodies in serum and dried blood samples from RSM01-101 clinical trial: RSM01 mAb candidate, ADA against RSM01, and RSV-neutralising antibodies. As antibodies including RSM01 are generally not expected to partition to the cellular component, concentrations measured in whole blood were expected to be numerically lower than concentrations in serum [22]. This is because for a fixed collection of blood with a fixed amount of antibody present, processing the blood to serum would retain nearly all of the antibody in the serum fraction while the total sample volume would be decreased by approximately the cellular fraction in the clot. With serum samples having the same numerator and a smaller denominator versus blood samples, the reported concentration should always be higher.

During assay development for all analytes, we were able to attain 100% recovery using standard buffers (detailed methods in Supplementary information and as described earlier by Terstappen et al. [27]). This complete recovery helped maintain assay sensitivity even while reducing the total sample size.

### RSM01 PK in serum and blood

**Fig. 1:**
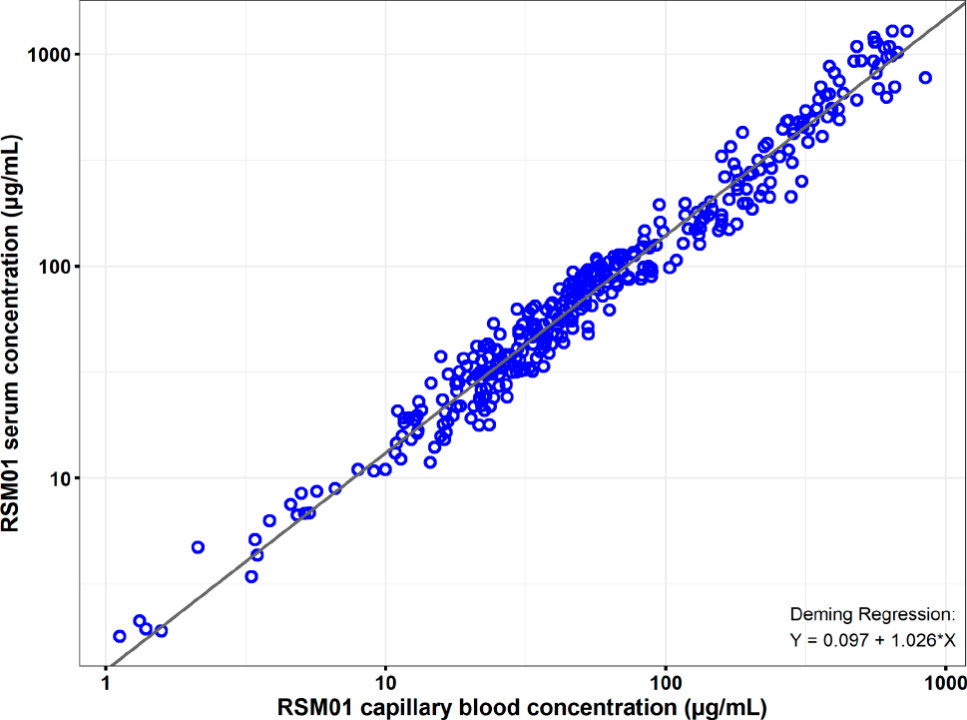
Deming Regression of RSM01 Concentration in Serum versus Blood. Log-log scale scatter plot of RSM01 concentration (µg/mL) for serum versus capillary blood. The Deming regression was performed with pooled data across all matching timepoints and from all cohorts, with variance ratio = 1 on log-transformed concentrations to yield the regression line of log (serum RSM01 concentration) = 0.097 + 1.026*(log_10_[capillary blood RSM01 concentration]). This regression line is shown in black

As previously seen for other monoclonal antibodies [17, 30], there was good correlation (R^2^ = 0.985) between individual concentrations of RSM01 measured in both serum and capillary blood (Fig. 1). In addition, the residuals were equally distributed above and below the regression line across the concentration range.

**Fig. 2:**
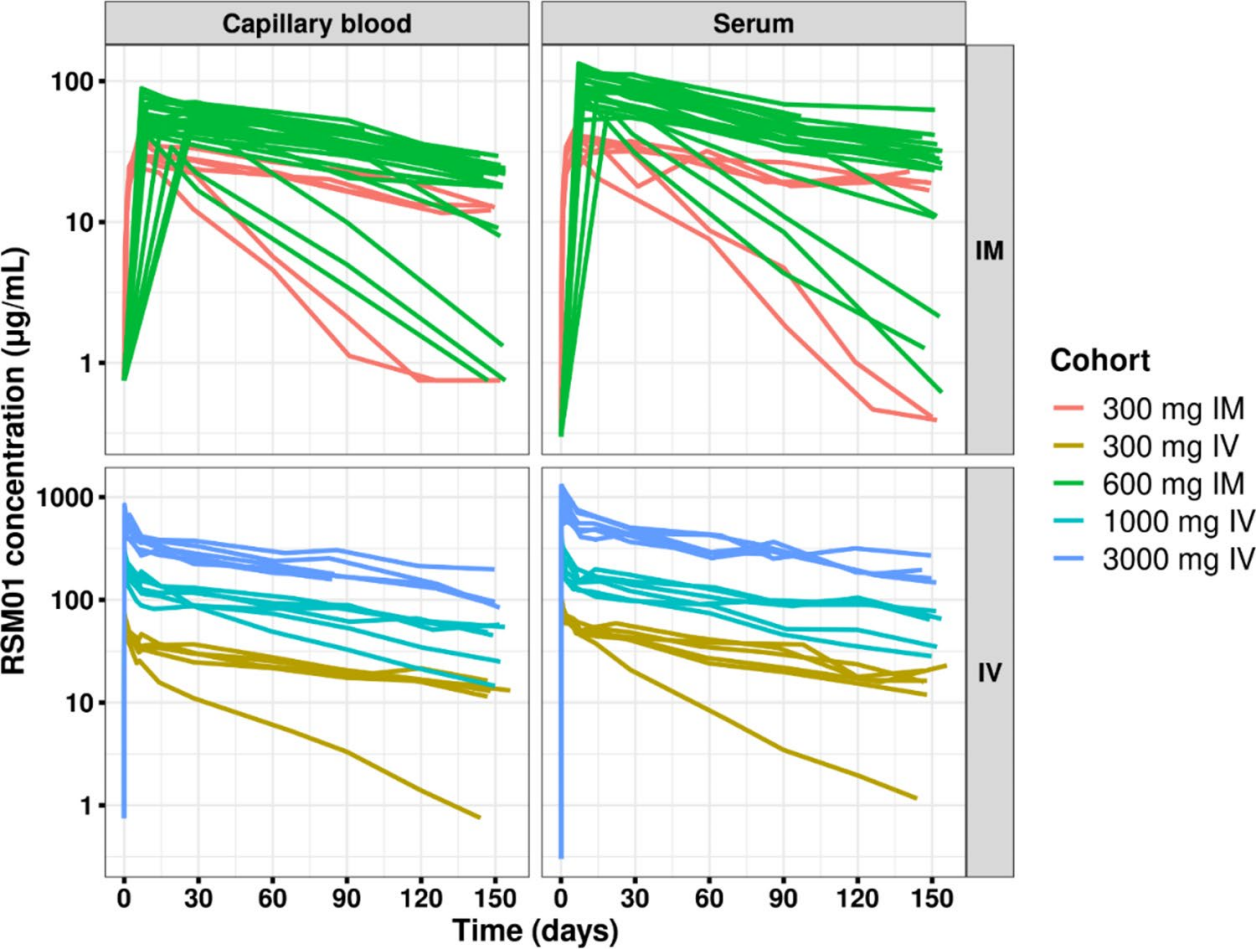
RSM01 concentration in serum and capillary blood over time by cohort, stratified by administration route The left column shows individual profiles derived from capillary blood concentrations and the right column shows individual profiles derived from serum concentrations. The top row shows individual profiles from IM administration and the bottom row shows individual profiles from IV administration

To further understand the impact on the specimen type on PK analysis, the non-compartmental analysis was conducted separately on both serum and blood data sets. Peak concentrations in capillary blood (T_max_) were reached between approximately 6 days and 8 days post dose for the intramuscular cohorts (Supplement Table 2). RSM01 was eliminated gradually, in a monophasic manner following IM administration and in a biphasic manner following IV administration (Supplement Fig. 1, Supplement Table 3). The observed between-participant variability was in line with the anticipated variability for a mAb following IM administration (Supplement Fig. 2). RSM01 concentration and AUC increased dose-proportionally following IV and IM administration with similar profiles across individual participants (Fig. 2). These PK results and conclusions are similar to those observed for serum samples [4].

The ratio of blood to serum PK parameters were performed for AUC_0 − D91_, AUC_0D151_, and C_max_ (Table 1). The geometric mean ratio was similar across all three parameters and cohorts.

**Table 1:** Ratios between blood PK parameters to serum PK parameters

Serum versus blood PK Parameters	Cohort 1 300 mg IV *N* = 6	Cohort 2 300 mg IM *N* = 6	Cohort 5 600 mg IM *N* = 24	Cohort 3 1000 mg IV *N* = 6	Cohort 4 3000 mg IV *N* = 6
AUC_0 − D91_	0.674 (14.9)	0.825 (8.9)	0.677 (13.2)	0.815 (10.3)	0.643 (10.6)
AUC_0 − D151_	0.703 (15)	0.797 (8.6)^*a*^	0.693 (13.4)^b^	0.783 (8.9)	0.665 (7.9)^c^
AUC_last_	0.700 (15.3)	0.776 (9.7)	0.690 (14.3)	0.783 (8.9)	0.653 (11.7)
C_max_	0.633 (16.7)	0.791 (17.8)	0.622 (15.3)	0.843 (19.8)	0.622 (22.1)

Summary statistics were performed on ratios from individual participants’ PK parameters ^*a*^*N = 5;*^*b*^*N = 23;*^*c*^*N = 4* Data are presented as geometric mean (CV%). *CV coefficient of variation; PK Pharmacokinetics*

### RSV-neutralising activity in serum and blood

**Fig. 3:**
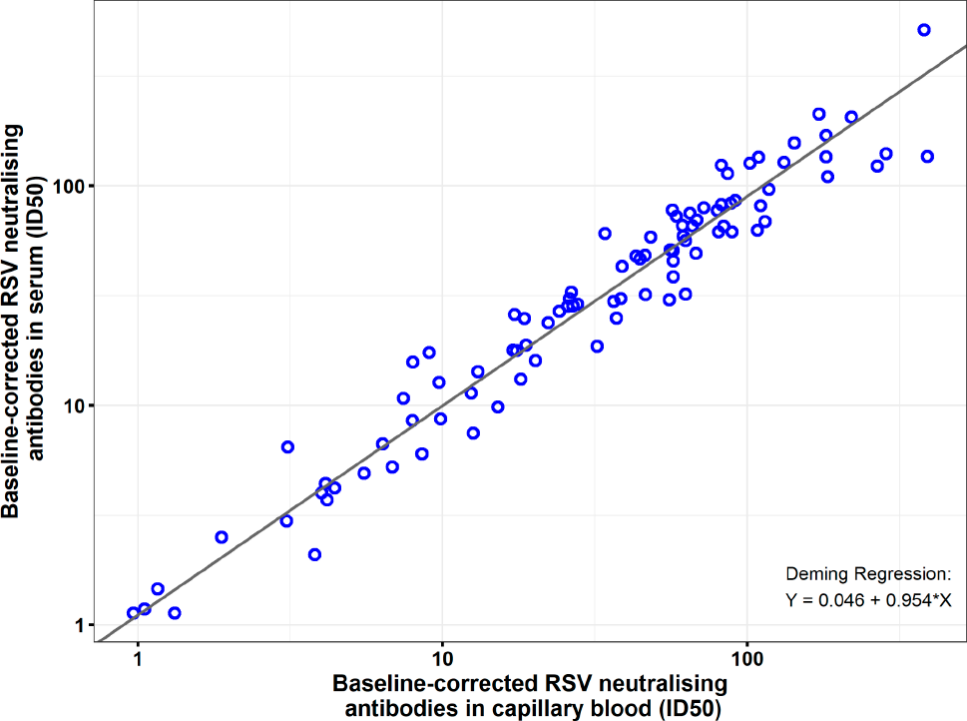
Deming Regression of RSV Neutralising Activity in Serum versus Blood. Log-log scale scatter plot of baseline-corrected RSV neutralising activity (ID50, reciprocal dilution corresponding to 50% inhibition of viral activity) in serum versus blood The Deming regression was performed with pooled data across all matching timepoints and from all cohorts, with variance ratio = 1 on log-transformed ID50 to yield the regression line of log_10_(serum RSV neutralising ID50) = 0.046 + 0.954*(log_10_[blood RSV neutralising ID50]). This regression line is shown in black

RSV neutralising activity was detected in all samples including prior to first dose for both serum and blood, with 10- to 100-fold increase over baseline at day 151 (Supplement Fig. 3). There was good correlation (R^2^ = 0.964) between the baseline-corrected RSV neutralising activity measured in serum and capillary blood (Fig. 3). In addition, the residuals were equally distributed above and below the regression line across the concentration range. (Note that the smaller n for baseline corrected ID50 reflects that all Day 1 values are normalised to 1 and therefore excluded from the correlation analysis.)

**Fig. 4:**
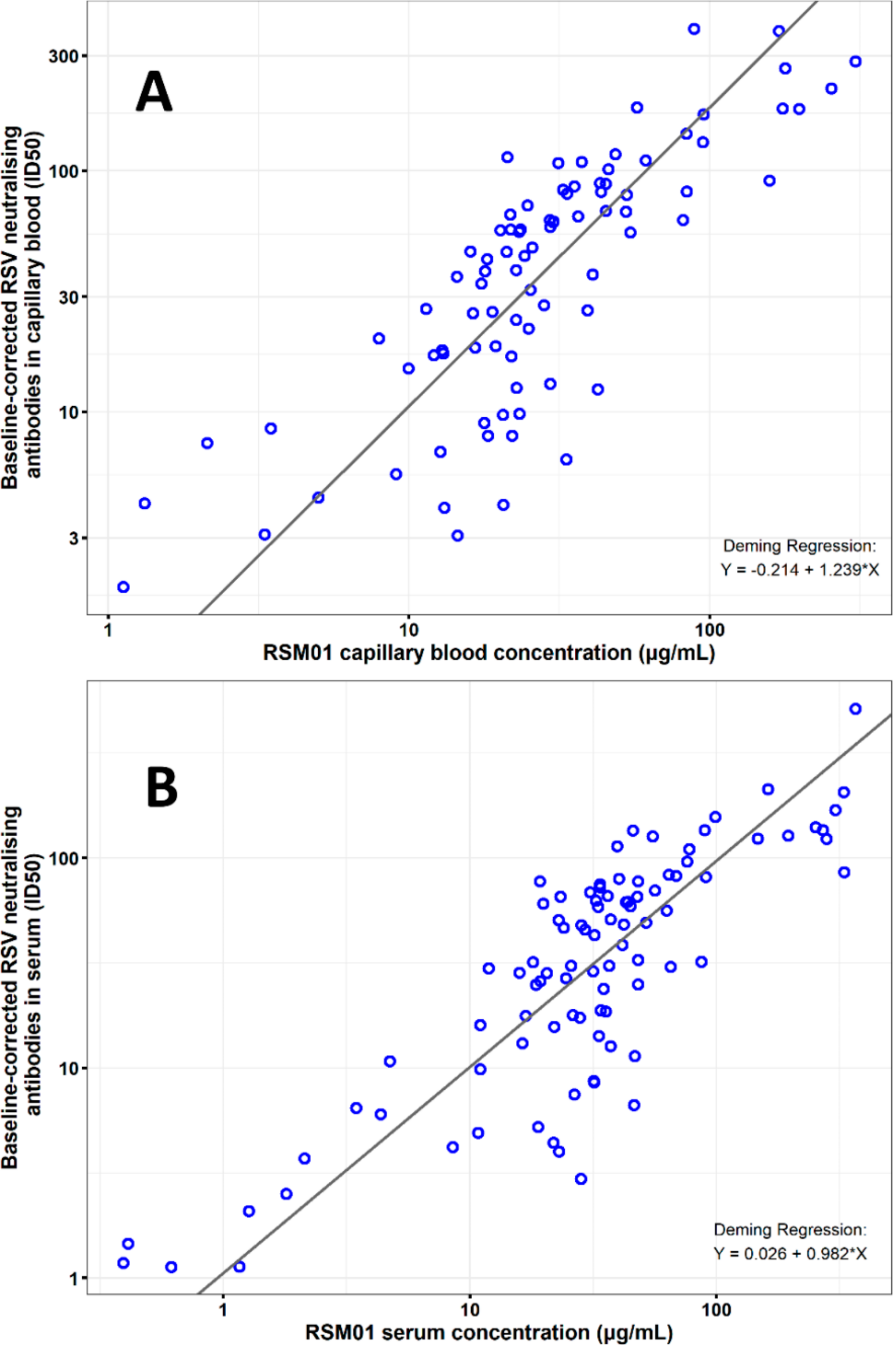
Deming Regression of RSV Neutralising Activity versus RSM01 concentration. Log-log scale scatter plot of baseline-corrected RSV neutralising activity (ID50) versus RSM01 (µg/mL) for blood **(A)** and serum **(B)**. The Deming regression was performed with pooled data across all matching timepoints and from all cohorts, with variance ratio = 1 on log-transformed RSV neutralising activity and RSM01 concentration to yield the regression lines of log_10_(blood RSV neutralising ID50) = -0.214 + 1.239*(log_10_[blood RSM01 concentration) and log_10_(serum RSV neutralising ID50) = 0.026 + 0.982*(log_10_[serum RSM01 concentration]). These regression lines are shown in black

To evaluate the relationship between RSV neutralising activity and RSM01 concentration, the Deming regression was performed for values derived from serum (Fig. 4A) and blood (Fig. 4B). As the RSV neutralising activity detects both RSM01 and any RSV neutralising antibodies from natural infection, there was no expectation that the values would correlate well with the RSM01 concentrations. While RSV neutralising antibodies generated by the participants’ adaptive immune systems may vary during the trial, a uniform baseline correction was performed within a participant by dividing post-administration ID50 values by their pre-dose baseline ID50. The Deming regression of baseline corrected RSV viral neutralising activity versus the RSM01 concentration showed a correlation between the two assessments over the range of values in both specimen types. While the regression was generally strong (R^2^ = 0.834 and R^2^ = 0.800 for serum and blood, respectively), the RSV neutralising activity exhibited proportionally more positive residuals at lower concentrations of RSM01 and more negative residuals at higher concentrations of RSM01.

### ADA in serum and blood

Because immunogenicity test results are not reported as continuous numeric data, the comparison was conducted by positive versus negative categorical results across both individual samples (Table 2) and overall immunogenicity status of subjects (Table 3). Individual sample results had 96% agreement (290/302) and overall participant status had 93% agreement (52/56).

**Table 2:** Agreement of serum and blood ADA results from individual samples

ADA in serum	ADA in blood	
	Positive^a^	Negative
Positive^a^	3 (1.0%)	4 (1.3%)
Negative	8 (2.6%)	287 (95%)

^a^Positive status includes positive samples with titre not reportable

**Table 3:** Agreement of overall participant status derived from serum and blood ADA sample results

Participant Status from Serum ADA results	Participant Status from Blood ADA results	
	Positive^a^	Negative
Positive^a^	1 (1.8%)	2 (3.6%)
Negative	2 (3.6%)	51 (91.1%)

^a^Positive status includes positive samples with titre not reportable

For serum measurements, the baseline ADA-positive rate was 2/48 (4.2%) with RSM01 [4]. The two baseline ADA positive participants were in the RSM01 600 mg IM cohort and one remained ADA-positive after RSM01 administration. One baseline ADA-negative participant in the RSM01 1000 mg IV cohort was categorized as postbaseline ADA-positive, for a treatment-emergent ADA-positive rate of 1/48 (2.1%) with RSM01.

For blood measurements, the baseline ADA-positive rate was 1/48 (2.12%) with RSM01. The baseline ADA positive participant was in the RSM01 1000 mg IV cohort and remained ADA-positive after RSM01 administration. Two baseline ADA-negative participants, one each in the RSM01 1000 mg and 3000 mg IV cohorts, were categorized as postbaseline ADA-positive, for a treatment-emergent ADA-positive rate of 2/48 (4.2%) with RSM01.

To better understand the potential source of discrepancies in ADA status, the ADA results from all individual samples for the 5 patients positive in at least one specimen type were examined. For the participant in the 1000 mg IV cohort characterized baseline ADA negative and post-baseline ADA positive in both serum and blood, seroconversion was detected at the same trial day with the same persistence of response with no discrepancies in individual results. For the 4 participants with discrepant overall status from discrepant individual sample results, the results were all close to the assay limit of detection which was established using an expected false positive rate (see Supplemental Methods).

The potential impact of immunogenicity on PK parameters was evaluated across both serum and blood data sets. In both specimens, concentration-time curves for participants who were ADA positive (either baseline or treatment-emergent) were within the limits of the other ADA negative participants in their respective cohorts [4] (Fig. 4).

## Discussion

Gates MRI RSM01-101 is the first-in-human, phase 1 trial of RSM01 mAb evaluating the safety, PK and immunogenicity of RSM01 in healthy adults [4]. In the present analysis, we evaluated the feasibility of using capillary blood instead of venous serum for bioassay measurements in future RSM01 clinical trials that will be conducted entirely in infants. We compared RSM01 PK, ADA, and RSV-neutralising antibodies between matched serum and dried blood samples collected in the RSM01-101 trial. Correlation between measurements in liquid serum versus dried blood was evaluated using Deming regression analysis.

Both RSM01 concentrations and RSV neutralising activity exhibited strong correlation between the serum and blood measurements. Lower concentrations and RSV neutralising activity were observed in blood compared with serum as expected, with antibodies primarily partitioning to the liquid but not to the cellular component of blood.

Notably, the RSV neutralising activity was retained in the cell-based neutralisation assay throughout the VAMS collection, processing, storage, and extraction steps. To our knowledge, this is the first published cell-based activity assay showing equivalent results for serum and dried blood. While we have only tested the blood extracts in one viral neutralising activity assay, we anticipate that retention of function would also be observed in other RSV neutralising activity assays employed for other mAbs and vaccines [31–35].

The PK analysis yielded similar conclusions in both blood and serum specimens. Variability within cohorts was similar. Dose-proportionality was observed in both data sets. While females have lower average haematocrit than males [36], no gender-based differences were observed in the correlation analysis or in pharmacokinetic analyses from either serum or blood.

Immunogenicity results from blood and serum specimens were also in high levels of agreement. It is difficult to determine explicitly whether the individual samples or categorization of the 4 participants with discrepant results are false positive or false negative. Certainly, the detection of ADA near the threshold is consistent with either hypothesis. Since both methods required use of non-parametric cut point factors, which are challenging to establish and highly dependent on the included samples, it is possible that this contributed to the Type I and Type II error rates in one or both assays.

Of note in this first-in-human trial in adults, we expected that the participants would have RSV neutralising activity at baseline from antibodies generated against prior natural infection. Therefore, baseline-correction was applied for comparison with measured RSM01. While this correction may still be required for future trials in infants with pre-existing neutralisation activity from maternal natural immunity, the baseline signal may not be constant over time as it depends on infant gestational age at birth, maternal natural immunity, and breastfeeding status [35]. For the infant population, the correlation with RSM01 will be evaluated with and without baseline-correction.

The correlation between RSM01 and RSV neutralising activity did exhibit bias as a function of RSM01 concentration. This bias was observed in both the serum and blood matrices, so does not disqualify either specimen collection. As this trial was conducted in healthy adults and overlapped with the COVID-disrupted RSV season, this bias may be due to the incomplete correction for natural immunity being more evident at lower RSM01 concentrations [37]. In the infant populations, the bias in neutralising activity versus RSM01 concentration may or may not remain. Additional hypotheses independent of trial population include bias of incurred samples in one or both assays which was not previously detected with spiked samples in validation, or an inherent non-proportional property of RSM01 in the viral neutralising activity.

The ratio of blood to serum may be lower and with greater variability in the infant population due to higher average haematocrit at birth that declines over the first 2 months of age [38, 39], although these trends are less dramatic in African infants [40, 41]. It is currently unknown whether haematocrit variability will meaningfully contribute to pharmacokinetic variability in infants. Use of a well-controlled immunoassay optimized and validated for blood measurements, a precise volumetric based collection device, and shipments at established stability conditions helps minimize preanalytical and analytical variability to better assess measures of biological variation. The potential contribution of haematocrit can then be evaluated by comparing Deming regressions and total variability from NCA derived from concentrations with and without haematocrit adjustment.

## Conclusions

For the RSM01 candidate, use of whole capillary blood sampled on VAMS is a suitable specimen type for the full clinical development program. Variability in blood was no greater for RSM01 concentrations, PK parameters, or RSV neutralizing activity than observed from serum. We encourage other sponsors to consider this approach to reduce the patient burden in clinical development programs. While the impact of reducing collection volume is greatest in the target infant population, other benefits of patient-centric sampling are broadly applicable, including streamlined processing at the clinical site and the potential to collect samples at locations with limited or no access to trained phlebotomists for venipuncture.

Implementing dried blood microsampling more broadly across clinical trials and post-marketing research on RSV prevention could help advance research into the most vulnerable patients who need these treatments, both premature infants and older adults with chronic medical conditions. Since these individuals also are at higher risk for difficult venous access [7, 8], these individuals may inadvertently be excluded from research into efficacy and safety clinical trials due to the inability to complete extensive venipuncture sample collection.

In addition, collection of microsamples during the registrational clinical trial enables direct translation to future sample self-collection by individuals in real world post-marketing trials, surveillance efforts, or even monitoring individual therapeutic or vaccine response to determine whether they need an additional monoclonal dose or vaccine boost. With both concentration and RSV neutralizing activity assays performing well in dried blood matrix, whichever measure is the best predictor of protection in the clinical trial can be measured from the self-collected dried blood sample.

While this manuscript focuses on implementation in the clinical trials, it is also possible to expand the blood microsampling into nonclinical research [42–45]. Implementation of this approach in nonclinical studies supports the 3R principles (Replacement, Reduction, and Refinement) [46] by enabling collection of exposure information in the pharmacology studies such as the cotton rat infection model.

As with every innovative approach, switching to patient-centric sampling approaches currently places more requirements on the sponsor including proactive engagement with trial investigators and institutional review boards, establishing a supply chain with the central laboratories for the nontraditional devices, education and training of clinical site personnel, and additional assay validation at analytical laboratories. Shifting the burden from the patient and site towards the sponsor early in development is anticipated to reap rewards in later stages of clinical development, including enabling hybrid and decentralized trial designs where biospecimen collection is required or adds scientific value.

## Electronic supplementary material

Below is the link to the electronic supplementary material.

Supplementary Material 1

## Data Availability

The Bill & Melinda Gates Medical Research Institute (Gates MRI; Cambridge, Massachusetts) is committed to data sharing that advances science and medicine while protecting privacy of trial participants. All summary datasets generated in the present analyses are available within the manuscript and its supplementary materials. Individual data points are not shared to protect participant data privacy. Other datasets used and/or analyzed during the current study are available from the corresponding author on reasonable request.

## Abbreviations

ADA: Anti drug antibodies
AUC: Area under the curve
DBS: Dried blood spot
ID50: 50% inhibitory dilution
IV: Intravenous
IM: Intramuscular
LMIC: Low- and middle-income countries
LRTI: Lower respiratory tract infection
mAb: Monoclonal antibody
MRD: Minimum required dilution
NCA: Noncompartmental analysis
PK: Pharmacokinetics
RSV: Respiratory syncytial virus
VAMS: Volumetric absorptive microsampling
