# Supplementary material for: Replacing serum with dried blood microsampling for pharmacokinetics, viral neutralisation and immunogenicity bioanalysis supporting future paediatric development of RSM01, a candidate respiratory syncytial virus neutralising monoclonal antibody
Source: BMC Infect Dis. 2024 Dec 18;24:1403. doi: 10.1186/s12879-024-10196-4 (PMC11653551; doi:10.1186/s12879-024-10196-4)
Supplement: Supplementary file 1 — Supplementary Material 1 [file 12879_2024_10196_MOESM1_ESM.docx]

# Supplement Methods

## Extraction from VAMS for measurement of RSM01 and ADA

The 20 µL VAMS tips supported in the Mitra 96-Autorack (Trajan Scientific) were placed in 200 µL of Extraction Diluent (1% bovine serum albumin (Sigma, Catalog# A3059) in 1x PBST (prepared from 20x PBST, Cell Signaling Technology Catalog# 9809)) within a 2 mL deep well polypropylene transfer plate. Samples were extracted at 5 °C overnight with 450 rpm shaking. After extraction, samples were centrifuged for 2 minutes at 500g to remove particulates from extract.

## RSM01 concentration

Calibrators for serum were made at 50,000, 25,000, 20,000, 10,000, 5,000, 2,500, 1,250, 625, 300, and 150 ng/mL RSM01 in pooled human serum, with the 50,000 and 150 ng/mL RSM01 as anchor points not included in the quantitative range. Quality controls (QCs) for serum were made at 18,500, 3,000, and 900 ng/mL RSM01 in pooled human serum. The lower limit of quantitation (LLOQ) in serum is 300 ng/mL.

Calibrators for VAMS were made at 50,000, 10,000, 8,750, 5,000, 2,500, 1,250, 800, 400, 200, 75, and 10 ng/mL RSM01 in 10% pooled human blood with K_2_ EDTA, with the 50,000 and 10 ng/mL RSM01 as anchor points not included in the quantitative range. QCs for VAMS were made at 7,500, 1,000, and 150 ng/mL RSM01 in 10% pooled human blood with K_2_ EDTA. Since these calibrators and QCs were made in the surrogate matrix of 10% human blood, the nominal concentration in human blood is 10-fold higher. The LLOQ for VAMS is 750 ng/mL.

Custom reagents for measurement of RSM01 were a monoclonal capture antibody and a conjugated detection antibody. The capture antibody was isolated from immunized mice with 2 rounds of subcloning to identify an anti-idiotype monoclonal antibody that bound RSM01 but not other human antibodies. The detection antibody was a goat-anti-human (Southern Biotech Cat# 2087-01) conjugated with a ruthenium tag (MesoScale Diagnostics Cat# R91AO).

MSD Standard Plates (MesoScale Diagnostics, Cat# L15XA) were coated with100 µL/well of 1 µg/mL capture antibody in 1x PBS (Fisher Cat# BP399). Plates were sealed and incubated overnight without shaking at 5 °C overnight. Plates were then washed 3x 300 µL/well with 1x PBST (Cell Signaling Technology Catalog# 9809) using automated plate washer (Biotek ELx405 or 405TS). Plates were subsequently blocked with 150 µL/well of PK Assay Buffer (5% bovine gamma globulin (Pel Freez Cat# 27005), 125mM NaCl (Fisther Cat#271), Blocker Casein (Thermo Cat# 37528)) for 1-3 hours at ambient temperature with 450 rpm shaking, then washed 3x 300 µL/well with 1x PBST.

Calibrators, two sets of 3 QC samples, and clinical trial samples (serum and extracted VAMS in separate assays) were diluted with PK Assay Buffer to the minimum required dilution (MRD) of 1:200. Each sample was transferred in duplicate for 50 µL/well to the coated and blocked MSD plate and incubated for 1 hour at ambient temperature with 450 rpm shaking. Plates were washed 2x 300 µL/well with 1x PBST, rotated 180°, and washed 2x 300 µL/well with 1x PBST. Then 50 µL/well of 0.25 µg/mL detection antibody in PK Assay Buffer was added and plates were incubated for 1 hour at ambient temperature with 450 rpm shaking. Plates were washed 2x 300 µL/well with 1x PBST, rotated 180°, and washed 2x 300 µL/well with 1x PBST. Then 150 µL/well of 2x MSD Read Buffer T (MesoScale Discovery R92TC) was added and the plate was immediately read on the MSD Sector 600 Imager.

RLU data were imported and analysed in Watson LIMS using a 5-PL fit with 1/Y^2^ weighting. Calibrators and QCs were analysed for % relative error (%RE) and precision (%CV). At least 75% calibrators within the quantitative range (6 of 8 for serum and 7 of 9 for VAMS) must have ±20.0 %RE and ≤20.0 %CV except at the quantitative limit of the calibration curve, the acceptance criteria are ±25.0 %RE and ≤25.0 %CV. For the QCs, 67% or 4 of 6 QC samples and at least 1 at each QC concentration must meet ±20.0 %RE and ≤20.0 %CV. RSM01 concentrations from acceptable runs were reported in ng/mL.

## Anti-drug antibodies

Custom reagents for measurement of ADAs were a positive control antibody and conjugated RSM01 for bridged detection. The control antibody was isolated from immunized rabbits with affinity purification against RSM01 and negative selection against human antibody. Two conjugates of RSM01 were prepared, the Bt-RSM01 was conjugated with NHS-biotin (Thermo Cat# A39257) and Ru-RSM01 was conjugated with ruthenium-based Sulfo tag (MesoScale Diagnostics Cat# R91AO).

Statistical thresholds were generated during validation using samples from 50 unique drug-naïve individuals after identifying and removing outliers and examining skewness of the data[1, 2]. The screening cut point factor (SCP) and titration cut point factor (TCP) were set at 5% and 0.1% false positive rates from negative individuals, respectively, and the confirmatory cut point factor (CCP) was set at a 1% false positive rate from negative individuals for percent reduction in excess RSM01. As the data was not normally distributed, the cut point factors were non-parametric established with the acquired data rather than a modeled normal distribution. For serum, SCP=1.50, CCP=37.3%, and TCP = 1.73. For blood, SCP=1.22, CCP=13.3%, and TCP=1.44.

Positive controls (PCs) for serum were made at 1,000 and 50 ng/mL positive control antibody in pooled human serum. The negative control (NC) is the same lot of pooled human serum without positive control antibody.

PCs for VAMS were made at 250, 100, and 20 ng/mL positive control antibody in 10% pooled human blood with K_2_ EDTA. Since these PCs were made in the surrogate matrix of 10% human blood, the nominal concentration in human blood is 10-fold higher. The NC is the same lot of 10% pooled human blood with K_2_ EDTA without positive control antibody.

QCs in duplicate and samples were diluted 1:10 with ADA Assay Buffer (1% bovine serum albumin (Sigma, Catalog# A3059) in 1x PBST (Cell Signaling Technology Catalog# 9809)) and then mixed in equal volumes with 2x Master Mix (4 µg/mL Bt-RSM01, 4 µg/mL Ru-RSM01 in ADA Assay Buffer). This is a net MRD of 1:20 for serum and 1:200 for blood, which corresponds to the minimum titre for each specimen type. These mixtures were incubated overnight at ambient temperature with 450 rpm shaking to allow the complexes to come to equilibrium, as only antibodies bound to one Bt-RSM01 and one Ru-RSM01 will be detected in the method.

MSD streptavidin coated plates (MesoScale Diagnostics Cat# L15SA) were blocked with 150 µL/well of ADA Assay Buffer for 1-3 hours at ambient temperature with 450 rpm shaking, then washed 3x 300 µL/well with 1x PBST. The samples were incubated overnight and transferred in duplicate with 100 µL/well to the blocked and washed plate, and then incubated for 1 hour at ambient temperature with 450 rpm shaking. Plates were washed 3x 300 µL/well with 1x PBST. Then 150 µL/well of 2x MSD Read Buffer T (MesoScale Discovery R92TC) was added and the plate was immediately read on the MSD Sector 600 Imager.

RLU data were imported and analysed in Watson LIMS as ratios to the negative control signal on the plate. The acceptance criteria for %CV of duplicate wells is ≤20%. The %CV criterion must be met for at least 50% at each PC level to accept the plate and the %CV of two NC sets must be ≤25%. For samples from accepted plates, a screen positive result was reported if the ratio to negative control was greater than the SCP.

Screen positive samples were assayed a second time in the confirmatory tier of the assay. Samples were assayed as described above plus a separate dilution in 2x Confirmatory Master Mix (4 µg/mL Bt-RSM01, 4 µg/mL Ru-RSM01, 50 µg/mL RSM01 in ADA Assay Buffer). Percent reduction of signal relative to the paired sample was calculated. A confirmed positive result was reported if the presence of excess unlabeled RSM01 reduced the signal more than the CCP.

Samples that confirmed positive were assayed a third time in the titration tier of the assay. Samples were assayed as described above except serial 2-fold dilutions of samples were prepared in serum or 10% whole blood prior to the MRD. The reciprocal of the highest dilution that yields a ratio to NC above the TCP is the reported titer. If the MRD yields a ratio to NC below the TCP, the sample is still considered positive, and recorded as titre not reportable (TNR).

## Selection of Deming Regression Method for Correlation Analysis

In ordinary Pearson linear regression, the focus is on minimizing the vertical distances between the observed data points and the regression line, assuming that only the dependent variable has measurement error, and the independent variable is known without error. However, in real-world scenarios including this data set, it is common for both variables to have measurement errors. The key concept behind Deming regression is to estimate the regression line that minimizes the total squared distances between the observed data points and the line. This method simultaneously accounts for the presence of errors in both the dependent and independent variables. The variance ratio, often assumed to be one as in this manuscript, is a parameter that specifies the relative variances of the errors in the dependent and independent variables. When this ratio is one, the errors in both variables are assumed to have equal variances, and the Deming regression is equivalent to an orthogonal regression. Overall, if there are errors in both variables, Deming regression provides a more robust approach to regression analysis compared to ordinary least squares regression. Due to the large range of reported values, we also performed Deming regression on log transformed data to account for variance from assay characterization representing a percentage of reported values rather than a fixed numeric value.

# References

1. Shankar G, Devanarayan V, Amaravadi L, Barrett YC, Bowsher R, Finco-Kent D, Fiscella M, Gorovits B, Kirschner S, Moxness M: **Recommendations for the validation of immunoassays used for detection of host antibodies against biotechnology products**. *Journal of pharmaceutical and biomedical analysis* 2008, **48**(5):1267-1281.

2. Devanarayan V, Smith WC, Brunelle RL, Seger ME, Krug K, Bowsher RR: **Recommendations for systematic statistical computation of immunogenicity cut points**. *The AAPS journal* 2017, **19**:1487-1498.

# Supplement Tables

**Table 1**: Sample collection schedule for matched serum and blood collection for RSM01, ADA, and RSV neutralising assessments

| Nominal sampling time | Analyte | | |
| --- | --- | --- | --- |
|  | RSM01 | ADA | RSV Neutralising |
| D1 Prior to first dose | Cohorts 1-5 | Cohorts 1-5 | Cohorts 1-5 |
| D1 (5 min) | Cohorts 1, 3, 4 |  |  |
| D1 (8 hr) | Cohorts 1-4 |  |  |
| D2 (24 hr) | Cohorts 1-4 |  |  |
| D3 (2 day) | Cohorts 1-4 |  |  |
| D6 (5 day) | Cohorts 1-4 |  |  |
| D8 (1 wk) | Cohorts 1-5 |  |  |
| D15 (2 wk) | Cohorts 1-4 | Cohorts 1-4 |  |
| D29 (4 wk) | Cohorts 1-5 | Cohorts 1-5 |  |
| D61 (2 mo) | Cohorts 1-4 | Cohorts 1-4 |  |
| D91 (3 mo) | Cohorts 1-5 | Cohorts 1-5 | Cohorts 1-5 |
| D121 (4 mo) | Cohorts 1-4 | Cohorts 1-4 |  |
| D151 (5 mo) | Cohorts 1-5 | Cohorts 1-5 | Cohorts 1-5 |

*ADA* anti-drug antibody, RSV respiratory syncytial virus
**Table 2**: Summary of RSM01 Blood and Serum PK Parameters

| **PK Parameters** | **RSM01 dose cohorts** | | | | | |
| --- | --- | --- | --- | --- | --- | --- |
| ***Blood*** | | | | | |  |
|  | Cohort 1 300 mg IV  N=6 | Cohort 2 300 mg IM  N=6 | Cohort 5 600 mg IM  N=24 | Cohort 3 1000 mg IV  N=6 | Cohort 4 3000 mg IV  N=6 | |
| AUC_last_ (h*µg/mL) | 68800 (54.9) | 50100 (63.4) | 115000 (49.6) | 279000 (27.8) | 705000 (29.0) | |
| C_max_ (µg/mL) | 62.2 (20.3) | 31.5 (17.6) | 56.4 (23.9) | 264 (18.2) | 652 (13.8) | |
| T_max_ (h) | 1.3  (1.25 - 9.28) | 140.46  (121.35 - 168.88) | 193.51  (165.77 - 695.38) | 1.81  (1.75 - 1.97) | 2.21  (2.18 - 10.17) | |
| ***Serum*** | | | | | | |
| AUC_last_ (h*µg/mL) | 98300 (43.4) | 64600 (56.0) | 167000 (40.7) | 356000 (26.6) | 1080000 (23.9) | |
|  |  |  |  |  |  | |
| C_max_(µg/mL) | 98.2 (15.4) | 39.8 (12.4) | 90.7 (21.6) | 314 (34.1) | 1050 (23.5) | |
| T_max_(h) | 1.25  (1.23 - 1.30) | 143.29  (121.53 - 670.42) | 173.18  (165.50 - 698.93) | 5.81  (1.73 - 25.70) | 6.19  (2.18 - 10.37) | |

All values are presented as geometric mean (geometric %CV) except for T_max_ which is presented as median (min, max). Individual values were presented when n = 1.

*AUC_last_* area under the curve from dosing to last measurable capillary blood concentration; *C_0_* initial concentration; *C_max_* maximum capillary blood concentration; *CV* Coefficient of variation; *D* Dose-Normalised; *IM* intramuscular; *IV* intravenous; *PK* Pharmacokinetic; *T_max_* time to maximum capillary blood concentration.

**Table 3**: Summary Blood Concentrations by Time Point and Cohort

| Time point (days) | Cohort 1 300 mg IV  N=6 | Cohort 2 300 mg IM  N=6 | Cohort 5 600 mg IM  N=24 | Cohort 3 1000 mg IV  N=6 | Cohort 4 3000 mg IV  N=6 |
| --- | --- | --- | --- | --- | --- |
| 0.03 | 62.52 (12.61) | - | - | 268 (46.25) | 655.83 (98.11) |
| 0.33 | 58.42 (8.02) | 4.22 (1.7) | - | 219.67 (34.16) | 596.33 (86.15) |
| 1 | 50.08 (5.5) | 12.02 (1.55) | - | 197.33 (37.7) | 505.17 (88.16) |
| 2 | 44.53 (3.89) | 21.48 (2.64) | - | 162.25 (22.2) | 516.2 (102.19) |
| 5 | 34.87 (6.74) | 31.42 (5.26) | - | 141.82 (25.52) | 411.83 (90.78) |
| 7 | 34.75 (6.81) | 31.23 (5.56) | 56.9 (14.17) | 140.12 (37.18) | 350.33 (53.46) |
| 14 | 31.6 (8.22) | 28.23 (4.21) | - | 118.77 (21.35) | 333.83 (41.01) |
| 28 | 27.37 (8.95) | 24.35 (7.07) | 50.16 (13.97) | 105.13 (19.77) | 296.2 (60.49) |
| 60 | 21.6 (8.29) | 17.24 (9.58) | - | 80.47 (18.47) | 218.33 (38.5) |
| 90 | 16.8 (6.74) | 13.12 (9.25) | 31.74 (12.73) | 66.82 (21.57) | 206.67 (59.07) |
| 120 | 14.51 (6.79) | 9.63 (7.26) | - | 49.32 (17.49) | 157.5 (37.7) |
| 150 | 11.47 (5.51) | 7.86 (6.5) | 18.68 (8.68) | 40.8 (17.18) | 118 (53.6) |

All values are presented as mean (SD). *SD* standard deviation

# Supplement Figures

**Figure 1:** **RSM01 capillary blood concentration over time after intravenous or intramuscular dose**


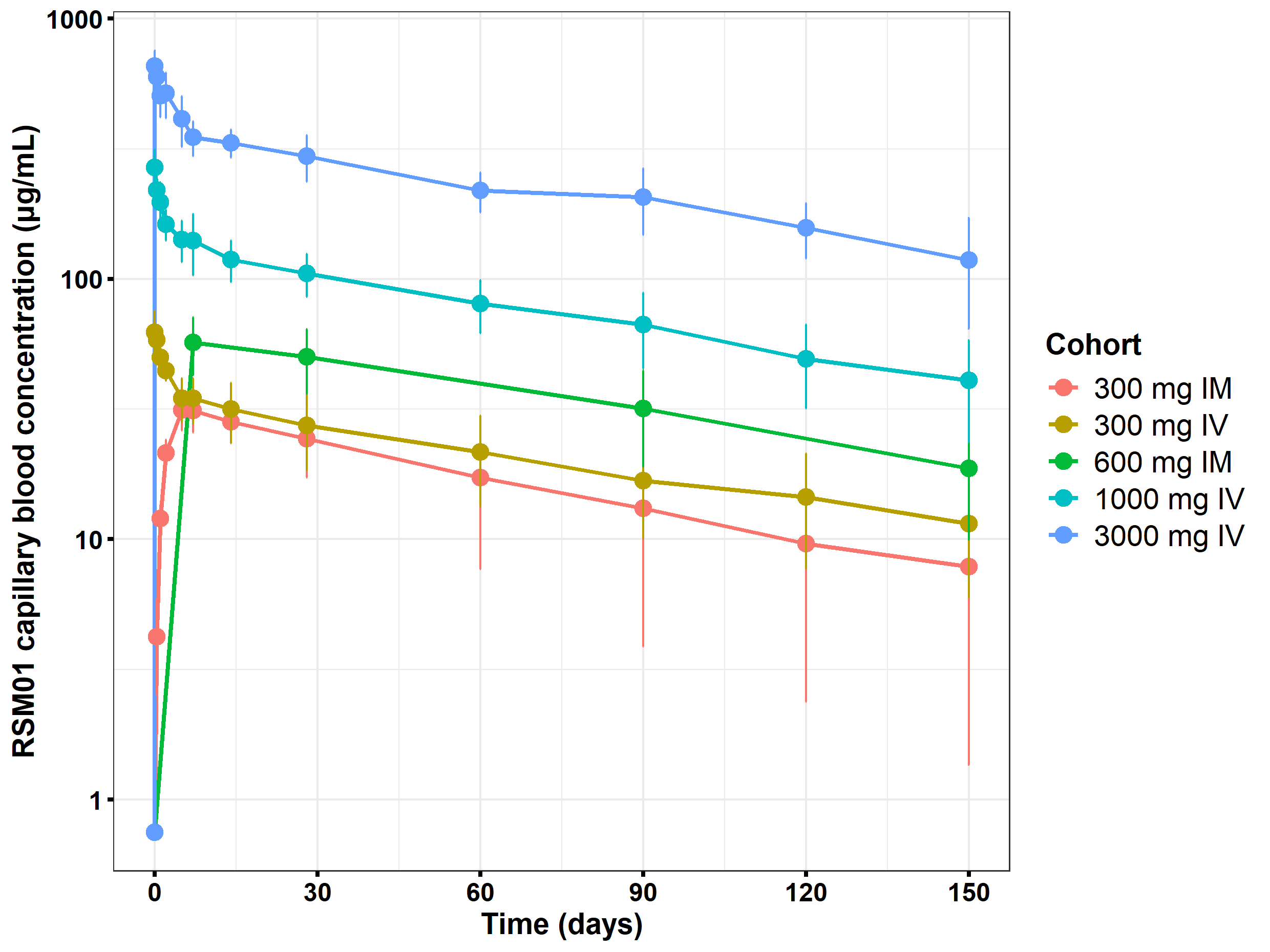


**Figure 2: RSM01 dose-normalised capillary blood concentration over time after intravenous or intramuscular dose**


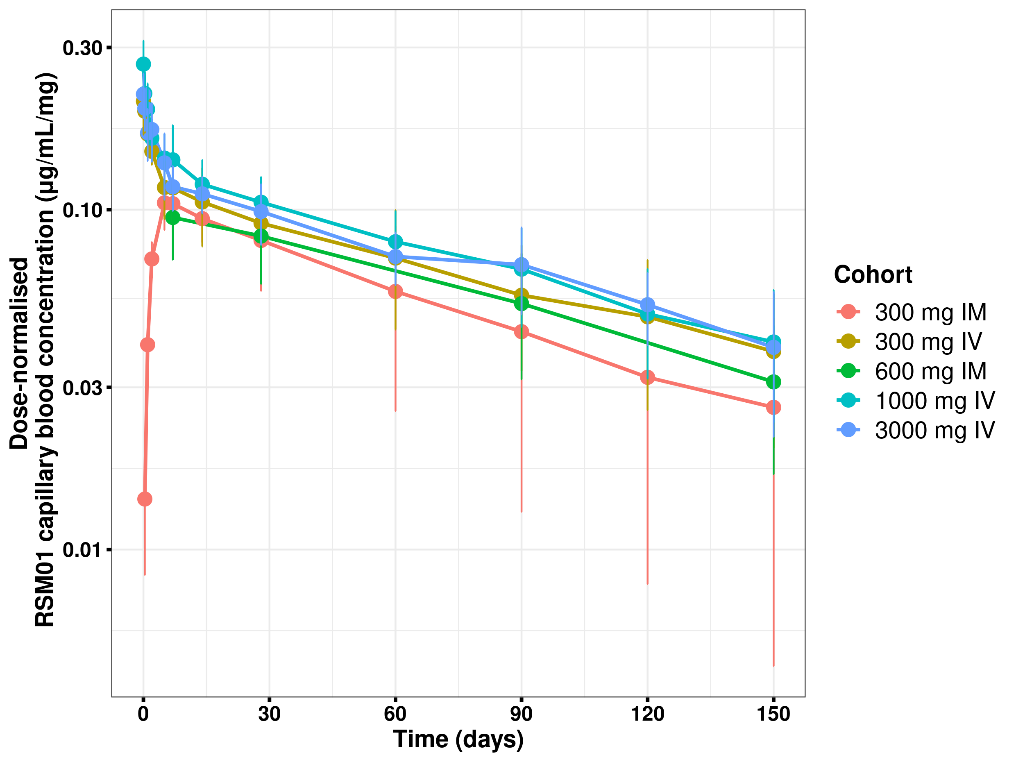


**Figure 3: Baseline-corrected RSV neutralising antibodies in capillary blood over time**


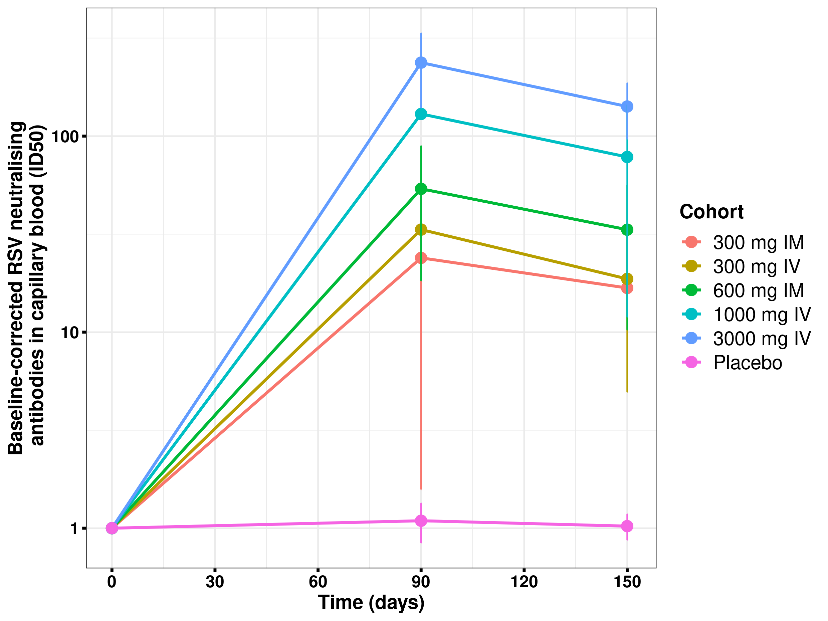


**Figure 4: Individual RSM01 capillary blood concentrations over time by ADA status, stratified by cohort**

**
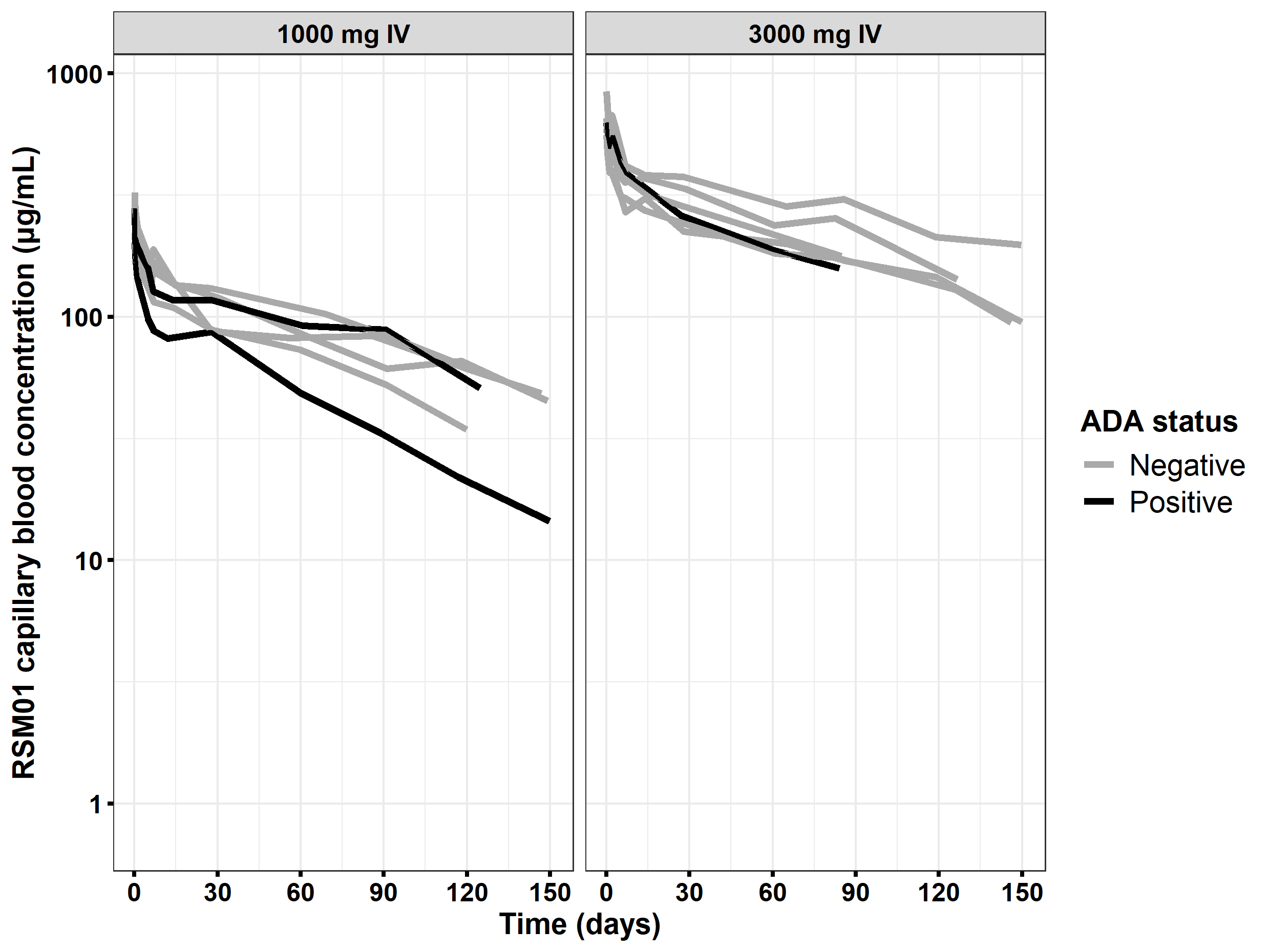
**
